# PTSD as a memory disorder

**DOI:** 10.3402/ejpt.v6.27633

**Published:** 2015-04-08

**Authors:** Hein van Marle

**Affiliations:** Department of Psychiatry, VU-Medical Centre and GGz inGeest, Amsterdam, The Netherlands

***Psychiatry as clinical neuroscience***: Adopting a cognitive neuroscience perspective on psychiatric disorders is crucial in attaining a deeper understanding of disease pathophysiology and symptomatology and developing novel theory-driven treatments. Worldwide, a great number of neuroscientists study basic brain functions like attention, fear, reward, and affect. The clinical disturbance of these brain functions is in part what we call psychiatry. Yet, most clinicians have very little knowledge of this literature, and intervention research in psychiatry is rarely guided by findings from fundamental neuroscience. In this presentation, I make the claim that post traumatic stress disorder (PTSD) can be considered a disorder of basic memory function. Hence, a more mechanistic account of PTSD symptoms, like flashbacks and hypervigilance, can be derived from fundamental insights into, for instance, the relationship between stress and memory and memory (re)consolidation. Furthermore, these findings (should) guide us in the development of novel treatments for PTSD.

***Memory disturbances in PTSD: focus on consolidation***: Although all phases of the memory cycle play their part in PTSD, the consolidation phase seems to be particularly important, and unlike the encoding phase (trauma acquisition), accessible to therapeutic intervention. During memory consolidation, initially fragile memory traces are reorganized and integrated into long-term storage (McGaugh, 2000). This process occurs when awake, but is particularly facilitated during sleep and can take up to years to fully develop (Diekelmann & Born, 2010). According to systems-level consolidation theory (Frankland & Bontempi, 2005), this reorganization of memory traces is realized through a gradual transfer of information from initial hippocampal-cortical links after encoding to predominant cortico-cortical connections, thereby effectively categorizing and conceptualizing the mnemonic episode.

On a neuronal level, this transfer is achieved through a process called replay during which the same hippocampal-cortical firing patterns that represent the stored memory during awake state are being played back to the cortex while asleep (Skaggs & McNaughton, 1996). This strengthens cortico-cortical associations and integrates the memory into existing cortical memory circuits. Currently, experimenters at the forefront of memory research aim to target this replay process in an attempt to boost memory consolidation. Successful attempts consist of presenting auditory or olfactory cues (sounds or smells) that were linked to memory encoding during subsequent slow wave sleep (e.g., Van Dongen et al., 2012).

In case of emotional memory, current theories suggest that memory consolidation additionally serves to preserve and solidify the declarative, factual aspect of an emotional memory trace, while at the same time depotentiating its affective charge (Walker & Van der Helm, 2009). This progressive decoupling of emotion and memory is thought to be facilitated by stress-related hormones and neurotransmitters (like cortisol and norepinephrine) that act at the level of the amygdala, thereby modulating the hippocampal-cortical transfer (Van Marle, Hermans, Qin, Overeem, & Fernandez, 2013).

***Understanding PTSD symptoms***: In case of traumatic memory as in PTSD, the process of memory consolidation seems to fail. The traumatic memory trace stays primarily located in subcortical and primary perceptual areas, leaving it tightly coupled to its autonomic and perceptual markers, and lacking the appropriate integration in autobiographical, cortical memory networks. Exposure to a trauma trigger subsequently results in a solely involuntarily retrieved memory trace (intrusion), that is very hard to verbalize, often fragmented in time, and consisting for the most part of primary sensory information (images, smell, sounds) that is linked to physiological fear symptoms (Brewin, 2011). Due to the lack of autobiographical context, the memory is relived as happening in the present. Thus a failure to properly consolidate and thus emotionally depotentiate potentially traumatic memories may form the neural basis of key PTSD symptoms like unwanted memories, intrusive flashbacks, nightmares, hyperarousal, and dissociation. Reduction of PTSD symptoms is accomplished by successful transfer to pre-existent, cortical memory circuits.

***From consolidation to reconsolidation***: An exciting development in memory research with clear implications for psychiatry has been the discovery of memory reconsolidation (Nader, Schafe, & Le Doux, 2000). In contrast to the idea that memory once formed is resistant to change, reconsolidation theory holds that after the retrieval or reactivation of a memory, it becomes labile and modifiable. This enables it to be reconsolidated in another form (updated) or prevented from being reconsolidated at all, effectively erasing it from memory. As opposed to memory extinction, in which a new (stronger) safety memory is formed in parallel to the original fear memory, this has the advantage of preventing the spontaneous recovery of fear. The initial experiment of Nader and colleagues showed that (toxically) blocking protein synthesis in the amygdala of the rat after reactivation of a conditioned fear memory led to its eradication from memory. Since then, an ongoing series of parallel experiments in rats and humans have found additional, more applicable methods of intervening in the reconsolidation of associative, aversive memory. Extinction learning itself can be used to update the original memory (Monfils, Cowansage, Klann, & LeDoux, 2009; Schiller et al., 2010), whereas propranolol (blocking noradrenergic action in the brain) disrupts the reconsolidation of original fear memories (Kindt, Soeter, & Vervliet, 2009), providing there is a form of prediction error during reactivation (Sevenster, Beckers, & Kindt, 2013). A critical time window of 10 min to up to 6 h after reactivation was found for all these interventions. Recently, Kroes and colleagues extended reconsolidation theory to naturalistic, episodic, emotional memory (Kroes et al., 2014). Implementing the appropriate control conditions, they showed that reactivation of a learned emotional storyline in depressed patients just prior to electroconvulsive therapy (ECT) treatment resulted in change-level memory of the story after 24 h. Hence, ECT seems to successfully prevent the restorage of emotionally laden information.

***Implications for (future) treatment***: Approaching PTSD as a memory disorder, with a specific focus on (re)consolidation opens up exciting, new ways to think about treatment. Current (pharmacological) intervention studies in PTSD however, in my opinion, fail to adhere to or be inspired by findings from (re)consolidation memory research. Several studies investigate the prolonged administration of propranolol or hydrocortisone for multiple days post-trauma (De Quervain, Aerni, Schelling, & Roozendaal, 2009; Pitman et al., 2002). Independent of the reported (mixed) effects, this leaves you uninformed about the mechanism of action because all phases of the memory cycle, including (spontaneous) retrieval, are affected. Alternatively, drug augmentation studies attempt to enhance the effect of exposure-based psychotherapy by administering drugs, mainly the NMDA receptor agonist d-cycloserine (DCS), in parallel to the therapeutic session. The mixed results (e.g. Litz et al., 2012; Rothbaum et al., 2014) are generally attributed to potentially also augmenting non-effective treatment sessions when the drug is administered prior to the session (Hofmann, Otto, Pollack, & Smits, 2015). Although this is theoretically correct, findings from (re)consolidation research emphasize the importance of timing drug augmentation to first post-session sleep (nap or night), thereby taking into account the critical time window of 10 min to 6 h after reactivation of the trauma memory. Alternatively to DCS, disruptive agents such as propranolol could prevent (as opposed to facilitate) restorage of the trauma memory when administered post-session. Invasive methods such as ECT or transcranial magnetic stimulation (TMS), when following reactivation, could potentially disrupt reconsolidation even more effectively, hypothetically erasing the trauma episode from memory.

Although often less accessible for therapeutic intervention, more research efforts should be directed towards intervening directly during post-trauma sleep, for instance, in emergency rooms or combat situations. The opportunity to influence the first-time transfer of potentially traumatic memory traces, either by administering drugs or just generally improving sleep quality, seems especially valuable.

A promising, but experimental, avenue for treatment is the selective targeting of replay to boost consolidation of trauma memory. Sound or smell stimuli that are linked to original trauma acquisition could be presented time-locked to different sleep stages or events. This patient-specific, trauma-tailored reactivation during sleep could by itself open up a new reconsolidation window, in which again facilitative or disruptive interventions can be applied.

So far, none of these interventions are available for clinical practice, but with a growing interest of clinical researchers in cognitive neuroscience, this is likely to happen sooner rather than later. A critical remaining question in PTSD pertains to whether any intervention aims to facilitate or speed up the (re)consolidation process thereby decoupling memory content from its affective charge (for instance, using DCS in first post-trauma sleep); or alternatively disrupt (re)consolidation of the traumatic memory trace thereby preventing its restorage (for instance, using propranolol or more invasive techniques such as ECT).
